# Primary Immunodeficiency with Severe Multi-Organ Immune Dysregulation

**DOI:** 10.1155/2019/8746249

**Published:** 2019-12-28

**Authors:** Tatyana Gavrilova

**Affiliations:** Assistant Professor, Albert Einstein College of Medicine, Attending in Medicine and Pediatrics, Division of Allergy and Immunology, Montefiore Medical Center, Bronx, NY, USA

## Abstract

Polyglandular autoimmune syndrome type 1, also known as autoimmune polyendocrinopathy-candidiasis-ectodermal dystrophy (APECED), is a rare primary immunodeficiency disorder with multi-organ involvement. Besides for being predisposed to severe life-threatening infections, patients with APECED are also prone to organ impairment secondary to severe autoimmunity. As this is an autosomal recessive disorder, a biallelic mutation in the *AIRE* gene is responsible for APECED. The author presents a case of APECED with a single *AIRE* mutation. Whole exome sequencing identified a mutation in the *BTNL2* gene that the author suggests may have contributed to the patient's presentation.

## 1. Background

Polyglandular autoimmune syndrome type 1, also known as autoimmune polyendocrinopathy-candidiasis-ectodermal dystrophy (APECED) syndrome, is a rare autosomal recessive disorder that presents with multi-organ involvement. Autoimmune disease is a hallmark of this disorder and its genetic etiology is a biallelic mutation in the *AIRE* gene that is responsible for immune regulation. The author presents a case of a patient with clinical APECED and a single pathogenic *AIRE* mutation. Whole exome sequencing identified a mutation in the *BTNL2* gene that codes for the butyrophilin-like 2 (MHC class II associated) protein and one that the author proposes may have contributed to his disease.

## 2. Case Description

A 15-year-old male patient presented with multiple autoimmune polyendocrinopathies (Tables 1 and 2). He had adrenal insufficiency, hypothyroidism, and hypoparathyroidism. He also had gluten-sensitive enteropathy and pancreatic insufficiency. The development of autoimmune hepatitis necessitated the initiation of systemic corticosteroids with a transition to oral tacrolimus 2 mg daily. The patient developed severe gastroparesis that required a gastrostomy-jejunostomy (G-J) tube in order to maintain appropriate nutritional support. He had recurrent nasal polyposis as well as one episode of an aural polyp in his left middle ear canal that required surgical resection. The patient developed Sjogren's syndrome, vitiligo, and candidal onychomycosis. He had recurrent infectious sinusitis and otitis, as well as more than ten episodes of pneumonia that required treatment with systemic antibiotics.

His mother is of Italian and Finnish heritage. His father is of Castilian descent. While his father is healthy, his mother has periodic fever syndrome, rheumatoid arthritis, Raynaud's disease, and hypoparathyroidism. The patient has one sibling, an older brother who has Raynaud's disease.

Flow cytometry quantified normal lymphocyte subpopulations (Table 3). Lymphocyte proliferation on mitogenic stimulation to PHA, ConA, and Pokeweed were within normal limits, as were antigenic responses to tetanus and candida. At the time of his evaluation he was already on immunoglobulin replacement therapy and his pretreatment immunoglobulin levels were not available. CD4+CD25+CD127^low^ populations were within normal limits making immunodysregulation polyendocrinopathy enteropathy X-linked (IPEX) unlikely. The clinical presentation was most consistent with APECED and genetic testing was therefore obtained.

Genetic sequencing identified a monoallelic pathogenic variant p.Arg257Ter in the *AIRE* gene at the c.769 C>T coding DNA. Further sequencing identified an exonic monoallelic frameshift mutation in the *BTNL2* gene at the enhancer coding sequence region 6:32370969 (TG>T), rs139418003 coding for p.His151ThrfsTer96.

## 3. Discussion

This case demonstrates a clinical phenotype of APECED but with a monoallelic pathogenic *AIRE* mutation. Further sequencing was therefore obtained in order to identify another possible contributing genetic factor.

The thymus is the site of T cell development and where central tolerance occurs. This organ is divided into the cortex and medulla, of which thymic epithelial cells (TECs) are a major component. The autoimmune regulator (AIRE) gene is critical for the development of the central T cell tolerance [1]. AIRE is a transcription regulator that is primarily expressed in the medullary thymic epithelial cells (mTECs), although its expression has also been identified in other peripheral organs such as the pancreas, testes, appendix, and adrenal cortex [2, 3]. mTECs express tissue-specific genes and tissue-restricted antigens (TRAs) that are presented to T cells within the context of major histocompatibility complexes (MHCs). AIRE regulates the expression of these tissue-specific genes and TRAs [3, 4]. High affinity T-cell receptor (TCR) interactions with MHC-presented self-antigens in the thymic tissue either induce the deletion of thymocytes by apoptosis or shift the T cells into regulatory T cell (Treg) development, thereby preventing autoimmunity [5, 6].

The severe autoimmunity that is seen in APECED is a result of defective AIRE function [7]. More than 100 mutations in the *AIRE* gene have been identified in autoimmune diseases. AntiIL17 autoantibody production, a cytokine that is important for antifungal immunity, has been identified in APECED. AIRE is also required for the function of the antifungal responses of Dectin-1 and Dectin-2 receptors [8]. These defects explain the predisposition of patients with APECED to developing candidiasis [9].

BTNL2 is a butyrophilin family member with homology to the B7 costimulatory molecules. Although its full immunoregulatory capacity has not yet been fully described, BTNL2 serves as a negative T-cell regulator by decreasing T-cell proliferation and cytokine release [10–12]. BTNL2 prevents B7-ICOS costimulation and downregulates T cell activation in vitro. Mouse studies have shown that recombinant mouse BTNL2 modifies B7/CD28 signaling to promote expression of Foxp3, a transcription factor involved in regulatory T cell development [13].

BTNL2 is highly expressed in lymphoid tissue. It has been localized to Peyer's patches of the small intestine as well as epithelial and dendritic cells within gastrointestinal tissue. Altered expression of BTNL2 is associated with inflammatory bowel disease [14]. Mutations in this gene are further associated with other autoimmune conditions including sarcoidosis, rheumatoid arthritis, vitiligo, and psoriasis [15–18]. BTNL2 has also been implicated in autoimmune thyroid disease [19, 20].

Given the location of the presently identified *BTNL2* frameshift mutation and its role in the regulation of T cell-mediated responses, the author proposes that this mutation may have contributed to the development of the severe autoimmune manifestations in this patient with a single pathogenic *AIRE* mutation. Continued reports of genetic polymorphisms of the *BTNL2* gene in the presence of autoimmunity will allow for a better understanding of the scope of this gene's impact on immunoregulatory pathways.

## Figures and Tables

**Table 1:** Chronological clinical features.

Condition	Age
Oral thrush	9 months
Pancreatic insufficiency	2 years
Adrenal insufficiency	3
Uveitis	4
Candidal onychomycosis	5
Enteropathy	6
Alopecia	7
Raynaud's disease	7
Vitiligo	8
Sjogren's syndrome	10
Hypoparathyroidism	11
Hypothyroidism	12
Gastroparesis	12
Nasal polyps, recurrent	12
Aural polyp	12
Megacolon	12
Autoimmune hepatitis	15

**Table 2:** Autoantibodies.

Antibody (units)	Results	Reference Range
ANA	negative	negative
DsDNA (IU/mL)	1.1	<70.1
Rheumatoid factor (IU/mL)	<9.9	0–22
Thyroglobulin (ng/mL)	0	0
Anti-TPO antibody (IU/mL)	567.2	0–5
Glutamic acid decarboxylase antibody (IU/mL)	>250	<5

**Table 3:** Lymphocyte subsets.

Lymphocyte	Percentage	Cells/*µ*L
T+B+NK+	79	2170
CD4+ T cells	46	1251
CD8+ T cells	26	720
CD19+ B cells	11	320
CD16+CD56+ NK cells	6	180
